# Venerable Men

**Published:** 1872-01

**Authors:** 


### HALL'S

### JOURNAL OF HEALTH.

VOL. XIX.—JANUARY, 1872.—No. I.

### VENERABLE MEN.

THEY live longest, as a class, who lead calm and even lives, mentally and physically; who are most exempt from the turmoils and shocks and strains which are incident to human existence, and who are assured of to-morrow's bread. There is no one thing, aside from the blessedness of an implicit reliance on the Providence of God, which has such a direct influence in promoting longevity as an assurance, felt to be well grounded, of a comfortable provision for life, for all the ordinary wants of our station. Not long ago a man died in a poor-house in England, where he had been taken care of for ninety years; he had no anxieties for to-morrow's bread; he had no quarter's day to provide against, in default of which wife and children would be turned into the street from the doors of the elegant brown stone mansion. He had no notes to meet in bank, which if not paid by a day and an hour would involve protest and financial ruin. Ah, this load of debt! how it grinds one's manhood to powder; how it agonizes the sensitive heart; how it shames a man's honor; how it has driven men to desperation, to drunkenness, to suicide, to murder! How the anguish of it takes the energy and health out of a man, and makes him pine and languish for weary days and weeks on beds of thorns, which pierce through the body, into the soul!

So, one good way to avoid sickness and premature death is to avoid debt as you would the devil. But see how the combined influences of calmness and goodness and pecuniary independence extend men's lives beyond fourscore, and even in sight of the century!

The Rev. Dr. DeWitt, of the Reformed Church, himself, his wife, his parish, all individually rich, has been preaching sixty years, and through all that time has maintained the love of his people and the confidence and respect of all New Yorkers, and at the age of eighty-two is loved and honored still.

Dr. Spring, of the Brick Church, is now eighty-seven years old, has been all his life "a working man," and one of the bulwarks of the Presbyterian Church, always well-cared for. And next is Samuel Hanson Cox; there is only one like him on the globe. No need of writing Reverend before his name, nor putting all the letters of the alphabet after it, to designate his degrees; at fourscore he is as lively as a young buck; as full of fun as of Latin, Greek, and Hebrew; and even now can out-preach and out-quote nine tenths of the dyspeptic young Reverends who swarm out of Princeton and Alleghany every summer. Then there is Father Cleveland, in sight of a hundred, still loving to preach the gospel; and Peter Cartwright, the great Methodist; and yesterday, we rode beside a man with as lively an eye as any girl of sixteen, of deep, deep blue, with faultless dress and courtly mien — Captain Lahebush — now in his hundred and sixth year. These men all, besides that religion which hath the promise of the life that now is, length of days in her right hand, have had one thing in common, — worldly competence; enough to keep them from worry, from anxious care about to-morrow. Peter Cartwright was not a millionaire, in the literal sense of the word; he did not exactly have a bushel of "five-twenties" in his iron safe; for, poor man, he never was worth an iron safe in all his life. All the money he has ever handled, of his own, would be but a fraction of the cost of one of the least that was ever burnt up at Chicago! The nearest approach he has to one is a Methodist circuit rider's pair of old leather saddle-bags; but they have carried his treasures safely for seventy years, to wit, his Bible and Hymn-book; these were his letters of credit, good at sight for all he wanted, and would be honored in the princely mansion of the Avenue, as well as in the hut of the forest or the hovel of the prairie. The observant know that paupers and pensioned live long; there are more gray-headed men in a city church than in a country meeting-house; more very old ladies. On inquiry it will found, in four cases out of five, that they are well provided for. Small wonder is it then that the sterling, practical common sense of the masses leads them to struggle to lay up something for a

RAINY DAY, that when they are old there will be a little ahead; something in the bank, or on bond and mortgage, or in national securities, which will relieve them from the necessity of working hard when they get old; making it a certainty that "their bread shall be given them, and their water shall be sure;" it is this assurance that gives a quiet mind, a composed spirit, and an infant's sleep, so sweet, precious, heavenly.

INVESTMENTS.

No man or woman ought to reach fifty years without having laid up some money, in some form or other, which will yield an interest, even if it be but a hundred dollars; then there is the sum of six dollars made every year without labor; made, rain or shine, summer or winter, whatever may be the crash of merchants and of men. If the amount be ten hundred or ten thousand dollars, so much the better. To invest that amount in such a way as to produce the greatest quietude of mind is worth knowing. A man said to me the other day, on inquiring how he invested his money, whether on notes, or mortgages, or railroad bonds: "Neither of them, Doctor. You knew me when I was first married; and my sweetheart's father refused to give his consent that I should have his daughter, because I had nothing ahead; but we married for all that. I am worth some half a million to-day, earned by my own individual efforts, within twenty years, in legitimate business; in doing this, I have had many dealings with many men; I have enough now. I want peace and quietude, and to be happy; but to be happy, I must have my time fully occupied, yet in such a way as not to be ruffled or annoyed. To bring about these results, I have placed my affairs in such a shape as to avoid any business transactions with men, except to pay for what I want on the spot; this I have done by purchasing government securities; they do not bring me as large an interest, by nearly one half, as I could get in various other directions, but I get it always at the day, with the utmost certainty. If I lent my money to a friend, he might not be ready to pay the interest at the time it was due. I would not want to press him; at the same time, having made my calculation to get it punctually, I might be put to some considerable inconvenience, and knowing that I could do without it, he might not put himself to special inconvenience; and before he knew it, another quarter's interest would be due. Or, he might be anxious to pay, and might be worrying himself half to death at his inability to pay, and this would disquiet me. If I loaned my money on

BOND AND MORTGAGE

to acquaintances or strangers, I would often be subject to their earnest desires for an indulgence, or to the necessity of listening to long rehearsals of their disappointments; the impositions which have been practiced on them; and to ruinous losses, having in perspective the necessity of their property being sold to pay the debt. As to railroad stocks and bonds, and insurance companies and banks, they are often mismanaged; their cashiers run away with the money; presidents of insurance companies use up the assets in paying themselves their own salaries, and banks are breaking all the time. Hence, looking over the whole ground, I concluded to invest in government securities. I do not get so much interest, but I get it certainly, without trouble and without annoyance."

Such were the reasonings of a successful business man and Christian gentleman. We leave the wisdom of his course to be decided upon by our readers each for himself. It might be inquired, May not the government fail? Yes; but the chances of failure are so much less than the failure of banks and companies and men, that it is scarcely worth while to take it into consideration.

The almost universal error of persons who have money to lend, is their placing it where it will yield the highest interest, forgetting that the higher the interest, the greater the probability of losing principal and interest both. It may be set down as a very general rule, that money lent at a high interest stays lent; or the interest is paid by piecemeal, and at uncertain intervals, making it impossible to place any reliance upon it, either as to the time or amount. Let it not be forgotten that a small sum, certainly and regularly received, is worth more than double the amount which is always behind the time, and comes in driblets; better a thousand times to take less, and arrange to live on that, than, on the mere promise of more, to live up to that promised income, and find yourself disappointed, and then have to disappoint others. It will be great wisdom in all persons who are living on an income, but a wisdom which not one in ten thousand possesses, to

LIVE BEHIND YOUR INCOME, instead of in advance of it; this can be managed by refusing to purchase, even to the amount of five cents, unless you can pay for it on the spot; then, when your quarter's income is paid you, the whole of it is in your pocket, and you do not owe a cent. But instead of this comfortable arrangement, the great mass of salaried persons and those who have a stated income, live in advance of it, and on interest day, every cent of it

IS BESPOKEN; and if they are honest, every cent is paid out on the self-same day, with the unfortunate result that they are always out of money, and

ALWAYS IN DEBT, with its infernal slaveries. Within a week I said to a friend, whom I knew had been making money largely, by the mere force of diligence in business of a legitimate character, a man whom in 1863 the war left without a business and without a dollar, "Now that you have money again, and do not owe a penny to any human being, go and buy a house and lot, and

GIVE IT TO YOUR WIFE.

She has made your home a blessing to you, has taken care of the children, and in doing so has worked hard from morning to night, while you were working, but no harder, in your place of business; hence it is as much hers as yours; and now you ought to divide the profits, and let her have her share as her right, to be her own individual property; and if reverses should come to you again, and the fire should strip you of all you had, then she would have a home to shelter you and yours."

"Why, Doctor, I did that long ago."

"Well then, go a little further; your profits, your clear profits amount to a good many dollars every day, and I know it has been the case for a long time, and as you do not speculate, you must have a goodly sum in bank. Let me persuade you to invest for her; and in case you should be disabled, or calamities come and leave you poor again, you would not only have a house and home to shelter you, but she would have an income, which would at least buy bread for you and the children."

"Why, bless your heart, Doctor, I placed ten thousand dollars to her credit in the savings bank, a year ago."

In the darkest days of thé war I was riding down Broadway in an omnibus, and an old friend entered, whom I had not met for a long time; in fact, I thought he had left the city, for he was struggling to get a foothold, and I supposed he had failed to do so. His countenance was downcast; there evidently was some trouble at his heart, and being so unusual with him, I said, "My good fellow, you are

DOWN IN THE MOUTH.

What's the matter now, burned out again?"

"No, Doctor, but wife was worried when I left home this morning, and when I know that is the case, I have no heart; it follows me all day, until I get back home again after dark."

We have thought of that a great many times since, and wondered how many such husbands there are in New York; a thousand? a hundred? a dozen? Now, as a general rule, New York husbands, we mean in the better walks of life, are a very indulgent and clever set of fellows; indulgent, many times, beyond their ability, making the necessities of their situation so imperative, that they have to apply themselves to business with an assiduity, and even a desperation sometimes, which compels them to regard their

WIVES' WORRIES

As too trivial to engage a moment's attention. Suppose Bridget does break the spout of a tea-pot, smashes a tumbler, burns a hole in the hearth-rug, forgets to say that the coffee is out until ready to sit down to the table, or to give information that there is not an ounce of coal, until after supper,—which things, by the way, Bridget seems to take a perfect delight in doing. What are these trifling annoyances to meeting a note in bank before three o'clock, when it is already past two, and not a dollar to meet it, because Jones failed to pay his bill, in consequence of Smith's failure to pay up, as he had not time to go to bank, because his "wife had a baby this morning?" Well, the wife couldn't help having the baby, for the time was up; and Smith couldn't be expected to be bothering himself about banks, when a little cherub had come to town, worth more than all the banks in the nation to him! and if Smith don't pay Jones, how can Jones pay the bank? and so it goes; it is a history many times repeated, every day of every month, from one year's end to another, in New York, in Philadelphia, in Boston, everywhere.

We have gone off in a tangent, have left the track, and, like an unfortunate "bolter" on the race-course, must go back to the place we started from, merely for the gratification it will afford the reader to be told that the man who could not attend to his business satisfactorily in New York when he had left his wife out of sorts in the country, several miles away, was the same man who was able to give that same wife a house and lot, paid for, and a credit of ten thousand dollars in a Broadway bank; showing among other things what a grand good thing it is to have a sympathizing husband, and a wife whose sterling worth, whose unswerving fidelity and affection, in the storms as well as the sunshines of business, compels that sympathy.

For fear the reader may not be able to see the connection of the various parts of this article, it may be necessary to recapitulate, concatenate, and frame together "the house that Jack built," into unified, consolidated, logical structure, thus wise: A calm and even life promotes longevity.

As most persons want to live long, and to be comfortable in their old age, — as religion and worldly competence are the certain means of securing the objects desired, — as all necessary worldly thrift attends those who are religious from principle and conviction, verifying these in a consistent life, every reader is counselled, this new month of a new year, which may be the last to many an eye which now runs over this page, bright though it be with life and health and hope, to "seek first the kingdom of God and his righteousness, and all these things shall be added unto you."

